# From biting to engulfment: curvature–actin coupling controls phagocytosis of soft, deformable targets

**DOI:** 10.64898/2026.01.28.702248

**Published:** 2026-01-30

**Authors:** Shubhadeep Sadhukhan, Caitlin E. Cornell, Mansehaj Kaur Sandhu, Youri Peeters, Samo Penič, Aleš Iglič, Daniel A. Fletcher, Valentin Jaumouillé, Daan Vorselen, Nir S. Gov

**Affiliations:** 1 Department of Chemical and Biological Physics, Weizmann Institute of Science, Rehovot, Israel; 2 Department of Bioengineering, University of California Berkeley; Berkeley, CA USA; 3 Department of Molecular Biology and Biochemistry, Simon Fraser University, Burnaby BC, Canada; 4 Department of Cell Biology and Immunology, Wageningen University and Research, Wageningen, the Netherlands; 5 Laboratory of Physics, Faculty of Electrical Engineering, University of Ljubljana, Ljubljana, Slovenia; 6 University of California Berkeley/University of California San Francisco Graduate Group in Bioengineering, CA USA; 7 Division of Biological Systems and Engineering, Lawrence Berkeley National Laboratory; Berkeley CA USA; 8 Chan Zuckerberg Biohub; San Francisco CA USA; 9 Department of Physiology, Development and Neuroscience, Downing Site, University of Cambridge, Cambridge, UK

## Abstract

Phagocytosis is a fundamental process of the innate immune system, yet the physical determinants that govern the engulfment of soft, deformable targets remain poorly understood. Existing theoretical models typically approximate targets as rigid particles, overlooking the fact that both immune cells and many biological targets undergo significant membrane deformation during contact. Here, we develop a Monte Carlo–based membrane simulation framework to model the interactions of multiple vesicles, enabling us to explore phagocytosis-like processes in systems where both the phagocyte and the target possess flexible, thermally fluctuating membranes. We first validate our approach against established observations for the engulfment of rigid objects. We then investigate how the mechanical properties of a soft target—specifically membrane bending rigidity govern the outcome of phagocytic interactions. Our simulations reveal three distinct mechanical regimes: (i) biting or trogocytosis, in which the phagocyte extracts a portion of the target vesicle; (ii) pushing, where the target is displaced rather than engulfed; and (iii) full engulfment, in which the target is completely internalized. Increasing membrane tension via internal pressure produces analogous transitions, demonstrating a unified mechanical origin for these behaviours. Qualitative comparison with experiments involving Giant Unilamellar Vesicles (GUVs, deformable microparticles) and lymphoma cells supports the relevance of these regimes to biological phagocytosis. Together, these results highlight how target deformability fundamentally shapes phagocytic success and suggest that immune cells may exploit mechanical cues to recognize among different classes of soft targets.

## INTRODUCTION

Fundamental biological processes, such as phagocytosis, involve cells adhering and engulfing external objects [1, 2]. This process has been extensively explored experimentally, and is known to involve the dynamic recruitment of the actin cytoskeleton which plays a crucial role in driving the growth of the membrane protrusion that wraps around the engulfed object [3–5]. However, how the actin recruitment and the resulting cytoskeletal force is coordinated with the membrane dynamics during this process is not well understood [6, 7].

We have recently shown that the actin coordination during phagocytosis of rigid objects can be explained very well by a theoretical model which includes a coupling between curved membrane protein complexes (CMC) that recruit and nucleate actin polymerization [8]. This model demonstrated that passive CMC can enhance the engulfment process, even in the absence of active forces (which represent the protrusive forces due to actin polymerization). Active protrusive forces that are recruited by the CMC enable even more robust engulfment, at lower adhesion energies and less sensitive to the object’s shape. Curved membrane proteins that recruit actin polymerization have been found experimentally to be associated with the leading edge of cellular protrusions [9, 10], which are involved in cellular adhesion, spreading and motility [11–13]. The theoretical model demonstrated that this curvature-actin activity coupling can explain many cellular shape and migration dynamics [14–16].

Motivated by these results, we explore here the interactions between a cell and a non-rigid object. We extend our model to allow for two vesicle-like surfaces to evolve and interact. The model predicts a spontaneous symmetry breaking between two symmetric vesicles containing passive CMC. For active CMC interacting with a soft passive vesicle, the model predicts different dynamic regimes which depend on the rigidity of the target: with increasing rigidity the active vesicle transitions from “biting”, to “pushing” and eventually “engulfing” the target. These transitions arise in this model purely from the physical interactions, and the curvature-force coupling. These theoretical results offer an explanation of recent experimental observations of immune cells interacting with artificial particles [17–19], vesicles [20] and cancer cells [21] of different rigidities, where both engulfment (phagocytosis) and biting (trogocytosis [22]) were observed.

## THEORETICAL MODEL

**FIG. 1.**
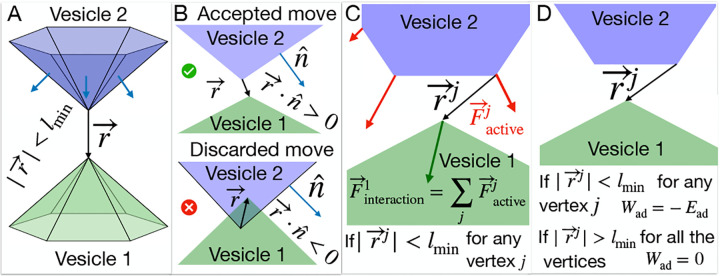
Interaction between two vesicles in the model: A) Two surfaces of two vesicles are shown in green and blue colors respectively. We check the distance between the vertices that belong to the two different vesicles and determine it they are interacting if the distance between them is less than the length unit of our simulation, i.e., $l_{\text{min}}$. B) If the distance $\left|\vec{r}\right|$ is less than $l_{\text{min}}$, we find the dot product between the vector $\vec{r}$ and the normals of all the triangles common to the vertex from the other vesicle $\overset{ˆ}{n}$. If the dot product is negative, the MC move is discarded as the vesicles are overlapping; otherwise, it is accepted. C) The interaction force on the vertex of interest is the vector sum of the active forces applied by the vertices on the other vesicle within the interaction range. D) If a vertex from vesicle 1 is at a distance less than the interaction range $l_{\text{min}}$ from any vertex of another vesicle, then the adhesive energy between them is $-E_{\text{ad}}$ for both of these vertices. Otherwise, it is zero.

Our theoretical model is based on the Monte-Carlo (MC) calculation of the dynamics of a closed three-dimensional triangulated self-avoiding vesicle with a spherical topology (Fig.1) [14, 23, 24] (See SI sections S1–S3 and Method and material for details). Within this model we denote the bare membrane nodes in blue, and the nodes containing the CMC in red. The active protrusive forces, representing the result of actin polymerization, are applied at the locations of the CMC. Our model is purely a membrane model, and it does not include any information about the details of the actin network inside the vesicle or the internal structure of the cell, such as organelles (nucleus) or bulk elastic deformations and their associated energy cost.

Here we developed our previous model to allow for the interaction between two dynamic vesicles (Fig.1). As a first step, we find the nodes that are adjacent between the two vesicles (Fig.1A). Such proximal nodes are restricted in their MC moves, as we do not allow the two vesicles to pass through each other. We need to check any such pair of vertices from different vesicles (Fig.1B, Fig.S1).

Next, we consider the active forces that a vesicle exerts on its neighboring vesicle. Any vertex in vesicle 1 feels the active force due to the other vesicle’s active CMC sites, that are within the interaction range (Fig.1C). The effect of this active force is included by adding the energy term given by,

(1) $$W_{\text{int}}=F\sum_{i}{\overset{ˆ}{n}}_{i}\cdot \vec{dr}$$

where i runs through all the vertices belonging to vesicle 2 within the distance of $l_{\text{min}}$ from the vertex of interest in vesicle 1, the force vectors have amplitude F and are directed at the outwards normal ${\overset{ˆ}{n}}_{i}$ at the sites i, while $\vec{dr}$ is the MC move of the vertex in vesicle 1. Note that in our model we do not explicitly maintain force balance. When adhered to external surfaces, they serve as momentum sinks, while for free vesicles we work in the center-of-mass frame which allows us to calculate relative shape changes, such that globally the force is effectively balanced.

Finally, we introduce an adhesion energy between proximal vertices on the two neighboring vesicles. Each vertex j that is within the adhesion range to the other vesicle (Fig.1D) has an adhesion energy that is given by,

(2) $$W_{\text{ad}}^{j}=-E_{\text{ad}}$$

Initially, we evolve it for a few Monte-Carlo steps from a pentagonal-dipyramid, until it becomes nearly spherical. The active force (when implemented) is set to $F=2k_{B}T/l_{\text{min}}$ and the protein-protein interaction energy is set to $w=1k_{B}T$ throughout the paper.

The area of the vesicles in our model is roughly conserved, with the bond lengths limited in their allowed range of fluctuation ($l_{\text{min}}<l<1.7l_{\text{min}}$) [23]. The typical changes in area in our simulations (without applied osmotic pressure) are of order 5% (see Fig.S7). When the osmotic pressure is applied inside the vesicle, the bond lengths are pushed towards their maximal allowed value (and therefore the average area increases), and the fluctuations in area are greatly suppressed.

## RESULTS

### Spontaneous symmetry breaking of adhering vesicles containing CMC

We first analyzed the adhesion between two identical vesicles, in the absence of active forces (F=0). For calibration purposes, we tested our model for adhesion of bare membrane vesicles (no CMC) and under the conditions of volume conservation (Figs.S2–S4, See SI section S6), for which analytic solutions are available [25]. In this limit, we could extract the contact angles between the two vesicles, and compare them to an analytic result [26–28]. The agreement between the simulations and the analytic calculation serves to validate our numerical procedure. Adding CMC induces larger spreading and some breaking of the rotational symmetry, though the two adhering vesicles remain symmetric with respect to each other (Fig.S4). In the presence of CMC the contact surface between the vesicles is not anymore composed of flat or spherical surfaces, as was found for simple adhered vesicles [25, 29].

**FIG. 2.**
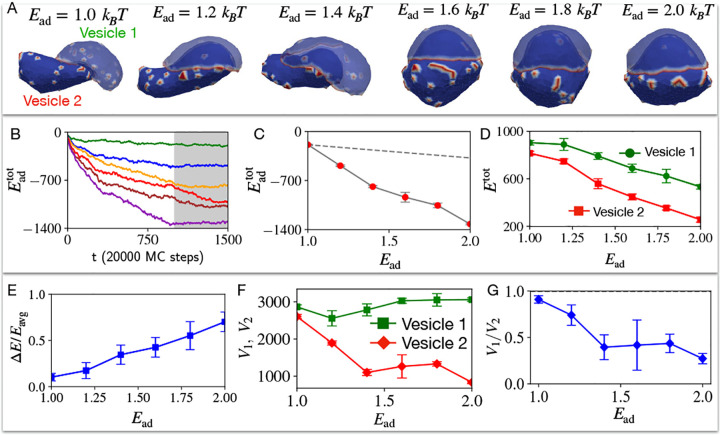
Two identical vesicles adhere to each other for different strengths of adhesion energy parameter $E_{\text{ad}}$. A) Final configuration snapshots of the pair of passive vesicles (at time step t=1500), for the adhesion energy parameter $E_{\text{ad}}=1,1.2,1.4,1.6,1.8,2.0$ in units of $k_{B}T$. Blue denotes the bare membrane nodes, while red at the passive CMC nodes. B) The average adhesive energy $E_{\text{ad}}^{\text{tot}}$ per vesicle is shown as a function of timestep for $E_{\text{ad}}=1,1.2,1.4,1.6,1.8,2.0$ in units of $k_{B}T$ with green, blue, orange, red, brown and purple solid lines respectively. C) The final total adhesive energy $E_{\text{ad}}^{\text{tot}}$ (averaged over the grey shaded time window shown in (B)) is shown for six different values of $E_{\text{ad}}$. The grey dashed line represents the total adhesive area in the case of $E_{\text{ad}}=1\;k_{B}T$ multiplied by the Ead. It shows that the total adhesive energy increases due to the increase in adhered area, faster than the increase in $E_{\text{ad}}$ (dashed line). In D) we show the total energies the pair of vesicles, averaged over the grey shaded region for six different values of $E_{\text{ad}}$. E) The relative difference between the vesicles increases with $E_{\text{ad}}$. F) Average volumes of the vesicles as function of $E_{\text{ad}}$, and G) the corresponding volume ratio. We used 722 vertices for each vesicle, out of which 50 vertices represent the curved-protein complexes with intrinsic curvature $c_{0}=1\;l_{\text{min}}^{-1}$, i.e. the CMC percentage is ρ=6.93%. Here, volume is not conserved.

**FIG. 3.**
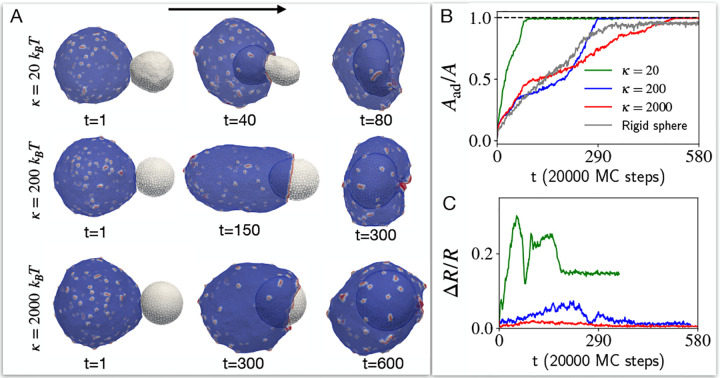
The engulfment of target vesicles of different rigidities by a cell-like vesicle with passive CMC. A) The snapshots of the shapes of the interacting vesicles with time for different bending rigidity κ values of the target vesicle. We set the bending rigidity of the cell-like vesicle at $20k_{B}T$. B) The time evolution of adhered area fraction of the target vesicle for different κ values (see SI section S4 for details on this calculation). As κ increases, the outcome is approaching the completely rigid κ=∞ limit that is shown in grey. C) Time evolution of the deviation of the target vesicle from a spherical shape, for different values of κ (color code as in (B)), measured by the relative standard deviation for the position vector of all the vertices with respect to the center of mass of the vesicle (Eq.S11). As κ increases, the target remains spherical all the time, and it takes longer time to engulf as it requires the cell-like vesicle to extend its adhesion cup over a bigger cross-section.

When we remove the volume conservation constraint (See SI section S7), we find that the presence of CMC can drive a spontaneous symmetry breaking above a critical value of the adhesion energy (Fig.2A). At low adhesion strength the two vesicles are still symmetric (Fig.2A, $E_{\text{ad}}=1k_{B}T$), but above a critical adhesion strength one of the vesicles spontaneously forms a cup-like shape (top vesicle in Fig.2A, vesicle 2), which partially encapsulates the other vesicle (bottom vesicle in Fig.2A, vesicle 1). The CMC in the top vesicle condense along the sharp rim of the cup shape, similar to the organization of such a vesicle when engulfing a rigid sphere [8] (Fig.3A). The bottom vesicle remains largely spherical, with the CMC randomly spread as small isolated clusters. This transition is driven by a lowering of the total energy of the two vesicles. The bending energy increases during the symmetry-breaking transition (Fig.S5), as the cup-shaped vesicle 2 is highly curved. However, this increase is offset by the adhesion energy between the vesicles which decreases the total energy (Fig.2B–E), and to a much smaller amount by the CMC-CMC binding energy (Fig.S5). The huge changes to the volume of the cup-shaped vesicle 2 during this spontaneous shape transition are quantified in Fig.2F,G.

Shapes that are similar to the vesicle pair of Fig.2(A) were obtained for adhering soft tissues (modeled as vesicles), where the symmetry breaking was induced by a large difference in the active surface tension between the two vesicles [30]. In our system the symmetry breaking is spontaneous, and the two vesicles have identical properties.

### Rigidity-dependent biting, pushing and engulfing

Next we explore the process of engulfment that mimics the phagocytosis of a non-rigid object by a cell. The engulfed (target) object is described by a small vesicle ($N^{T}=847$ vertices, forming a spherical object with radius of 10 $l_{\text{min}}$), and the cell-like (bigger) vesicle has 3127 vertices. We set the CMC concentration ρ=4.8% on the cell-like vesicle throughout this work. We start by validating our computation, demonstrating that for a very rigid target vesicle (bending modulus $\kappa =2000\;k_{B}T$) the engulfment proceeds in the same manner as we previously computed for a perfectly rigid sphere (Fig.S6, and see SI section S8) [8]. Note that by increasing the bending modulus we rigidify the target vesicle against shape deformations that increase local mean curvature, but explicit membrane tension and area fluctuations are unaffected.

We start exploring how a cell-like vesicle that contains passive CMC (F=0, Fig.3A), engulfs a target vesicle of different bending modulus. We find that the cell-like vesicle with passive CMC is able to fully engulf the target vesicle, with the engulfment proceeding faster for the softest target vesicle (See Movie S1). This faster engulfment is facilitated by the large deformation (See SI section S9 for details of deformation measurement) of the target vesicle (as shown in Fig.3B,C, Fig.S7B), which enables the cell-like vesicle to extend an adhesion cup over a smaller cross-sectional area.

**FIG. 4.**
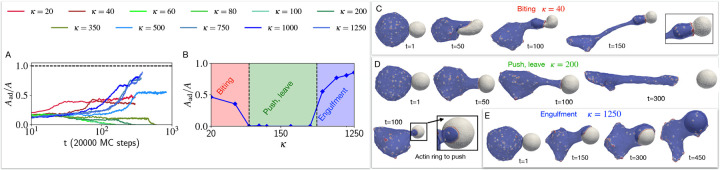
Effect of the bending rigidity of the target vesicle on the process of phagocytosis. The time evolution of the adhered area fraction of the target cell is shown in (A), for different bending modulus values. B) The final adhered area fraction of the target vesicle is shown as a function of the bending rigidity κ. We indicated three phases of biting, push-leave, and engulfment with red green and blue as κ increases. The time evolution of the shapes and the snapshots are shown for three example of biting, push-leave, and engulfment in C), D) and E) panels, where the bending rigidity κ for the target vesicle is set to $40\;k_{B}T$, $200\;k_{B}T$, and $1250\;k_{B}T$ respectively. We set the bending rigidity of the cell-like vesicle to $20k_{B}T$.

This behaviour is drastically changed when the CMC induce active protrusive forces. We explore in Fig.4 the engulfment dynamics as function of the bending modulus κ of the target vesicle, and find three main dynamical phases. We start with the high κ regime (blue traces in Fig.4A, and blue region on the phase diagram Fig.4B), where the cell-like vesicle completely engulfs the rigid target vesicle (similar to the engulfment of a rigid object, Fig.S6 [8]), as shown in the snapshots of Fig.4E. For a softer target vesicle (green traces in Fig.4A, and green region on the phase diagram Fig.4B), we find that the target vesicle ends up being pushed away (Fig.4D), and the contact area stalls in a “suction-cup”-like shape, and later retracts (Fig.4A) until the two vesicles detach (zero final adhered area fraction, Fig.4B) due to the high bending energy of the elongated ”finger” attached to the target vesicle (Fig.S8, See SI section S10). At even lower values of κ (red traces in Fig.4A, and red region on the phase diagram Fig.4B) we find that the engulfed area stalls at a small value ($A_{ad}/A<0.5$, Fig.4A,B), corresponding to a ”biting”-type dynamics (Fig.4C). Note that we do not allow the vesicles to undergo fission, even when greatly deformed.

**FIG. 5.**
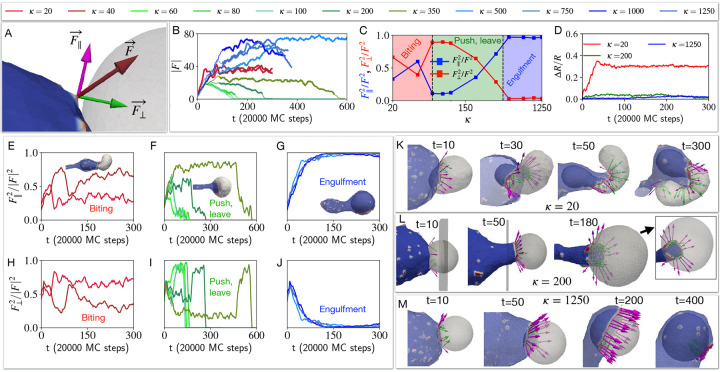
Coupling between the target vesicle deformation and alignment of the active forces during the engulfment process. A) The active force due to an active CMC node of the cell-like vesicle is applied to a neighboring node of the target vesicle $\vec{F}$. This force is decomposed into two parts, ${\vec{F}}_{\|}$ is tangential to the surface of the target vesicle and ${\vec{F}}_{⊥}$ is the pushing normal force (shown in magenta and green colour, respectively). B) The time evolution of the total magnitude of the force applied to the target vesicle by the cell-like vesicle is shown, for different bending rigidities of the target vesicle (as in Fig.4). C) Time averaged the tangential and normal force fractions as function of the bending rigidity of the target vesicle κ (over the times denoted by bold lines in (B)). D) The deviation of the target vesicle from a sphere is shown for three different bending rigidities κ of the target vesicle (Eq.S11). E)-G) show the fraction of tangential force on the target in the three dynamical regimes of biting, push-leave, and engulfment. H)-j) Similarly, the fraction of normal pushing force on the target in the three dynamical phases. K)-M) Snapshots showing the force decomposed into tangential and normal components together with the deformations of the target vesicle. We show how the early deformation caused by the normal components affect the later alignment of the CMC at the leading edge of the adhesion patch. K) A very soft target vesicle can initially adhere and bend into the cell-like vesicle, during the early stages. However, the CMC then impinge against the remaining target vesicle, and end up pushing and twisting it with a significant normal component. L) In the pushing regime the deformation of the target vesicle is sufficient to prevent the CMC from aligning tangentially, and a significant normal component maintains the pushing dynamics. M) For the rigid target vesicle the CMC cluster aligns tangentially and drives efficient engulfment. The bending rigidity of the bigger cell is set to $20k_{B}T$.

The origin of these dynamical phases can be understood when we investigate how the membrane shape and the orientation of the active forces are coupled. In Fig.5(A) we define the components of the active force that is exerted by a CMC of the cell-like vesicle when its in contact with the nodes of the target vesicle. This force is applied towards the outwards normal of the CMC, and has both normal ($F_{⊥}$) and tangential ($F_{\|}$) components with respect to the target vesicle node.

During the engulfment phase of rigid target vesicles we find that the CMC form large leading-edge clusters (large value of the total transmitted force, blue traces in Fig.5B) which mostly exert tangential forces (Fig.5G,J). This is shown in the snapshots of Fig.5M. The average fraction of the two force components (beyond the transient initial cup formation stage, denoted by the bold lines in Fig.5B), is shown in Fig.5C, and clearly demonstrate the relation between these force components and the resulting three dynamical phases.

In the pushing phase of slightly softer target vesicles we find that the leading-edge cluster gets arrested at a smaller size (green traces in Fig.5B), eventually disappearing when the two vesicles disengage. The dynamics of the force components (Fig.5F,I) show that the normal component remains relatively high, preventing the efficient spreading of the engulfing membrane over the target vesicle. In Fig.5L the snapshots show the origin of this behavior: due to the deformation of the target vesicle, the leading-edge of the engulfing membrane is not tangentially oriented, further pushing into the target vesicle and maintaining its deformation. This feedback between shape and force orientation arrests the spreading, as the target vesicle is pushed and deformed.

In the regime of softest target vesicle (red traces in Fig.5B) the target vesicle gets strongly deformed by the adhesion to and active forces exerted by the engulfing membrane (red line in Fig.5D). This large deformation prevents the CMC and the active force from aligning tangentially, with both components having similar magnitude (Fig.5E,H). Due to the large deformations of the soft target vesicle (Fig.5K) the leading-edge ends up mostly pushing it after a small portion is engulfed, leading to a ”biting”-like behavior. This is very different from the smooth engulfment observed when the active forces are absent (Fig.3).

The outcome of the engulfment process also depends on the environment, in the form of confinement and constraints on the membrane dynamics. As shown in Fig.S9, by holding a small patch of the target vesicle fixed in space, the outcome of the interaction can change from pushing to engulfment or biting (See SI section S11).

In Fig.S10 we show the same set of dynamic phases when we vary the osmotic pressure (see SI section S12 for details) inside the target vesicle (while keeping $\kappa =20k_{B}T$). We find that as the internal pressure p inside the target vesicle increases, the membrane of this vesicle gets stretched out and tense, inhibiting any shape changes and effectively stiffening the vesicle. Not surprisingly, the observed phases, as function of the pressure p (Fig.S10), perfectly match the phases observed as function of bending rigidity in Fig.4.

### Comparison to experiments

**FIG. 6.**
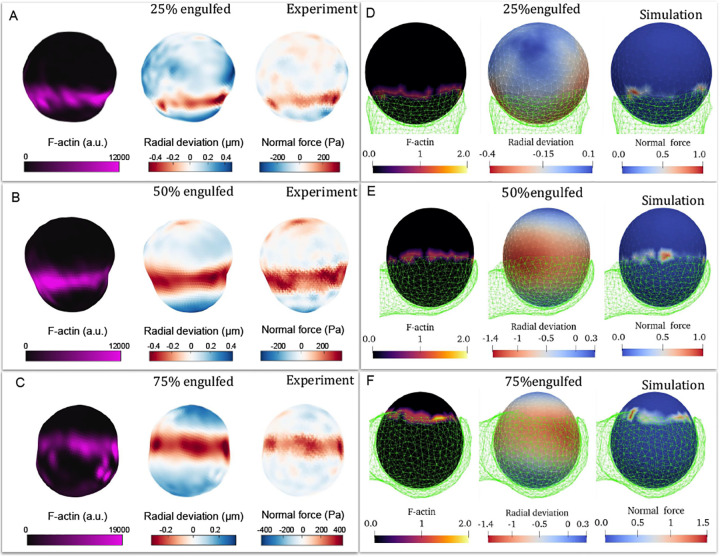
A-C) 3D reconstructions of deformable acrylamide-co-acrylic acid-microparticles (DAAM-particles) (1.4kPa,9μm) revealing F-actin over the particle surface, detailed target deformations induced during phagocytosis and normal forces inferred from shape deformations. DAAM-particles were functionalized with Immunoglobulin G (IgG) and stained with TAMRA-cadaverine and exposed to phagocytosis by RAW264.7 cells, which were then fixed and stained for F-actin. Each panel displays (left) F-actin distribution across the particle surface, (middle) radial deformation, and (right) the normal traction forces inferred from the shape deformations. (D-F) Snapshots of the simulated target vesicle (relatively rigid, $\kappa =750k_{B}T$) at different values of engulfed fraction (green transparent network denotes the engulfing cell-like membrane). Heatmaps show the location of the CMC (left), target vesicle radial deformation (middle) and the normal component of the active force exerted by the CMC on the target vesicle (right). These correspond to the actin localization in the experiments, bead deformation and normal elastic forces, respectively. The reconstructions in A, B and C correspond to the same engulfment percentages shown in D, E, and F, respectively.

We start by comparing the engulfment of a relatively rigid sphere ($\kappa =750k_{B}T$), in experiments utilizing elastic beads [19, 31] and simulations (Fig.6). Note that we are limited to relatively high values of κ in the simulations in order to ensure engulfment (Fig.4). In the experiments the actin recruitment is highly localized to the leading edge of the engulfing membrane, in agreement with our association of the actin force with the highly curved CMC cluster in the simulations. A band of normal force exerted by this leading edge on the engulfed sphere results in local squeezing of the sphere, in both experiments and simulations. The localization of the squeezing force and the shape deformation are more pronounced in the experiments compared to our simulations. This may arise due to the shell-like description of the engulfed sphere in the simulations, while it is a fully filled elastic bead in the experiments. There may also be additional contractile forces exerted by the cell at the leading edge, which we do not currently describe in our model. Nevertheless, the overall agreement is good.

Note that at the bead’s pole pointing at the engulfing cell the experiment measures a pulling force acting on the bead, which may arise from contractile forces pulling on these adhesion sites, or due to the effect of actin treadmilling emanating from the leading edge, exerting forces that pinch the bead at this pole (similar to actin treadmilling-induced forces that are involved with endocytosis [32]). This effect is absent from our simple model. We also observe that the actin at the leading edge is non-uniform and fragmented in both the experiments and our simulations, suggesting that a simple coupling between curvature and actin nucleation may be involved in a complex and non-uniform organization of this moving front [33, 34].

Experiments found lower rates of successful engulfment for soft beads [17, 19], in agreement with our simulation results (Fig.4).

**FIG. 7.**
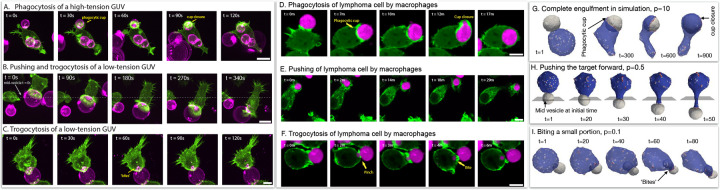
(A) Giant unilamellar vesicles (GUVs) composed of POPC and biotin-DOPE and filled with a hyperosmotic sucrose solution (300 mOsm), leading to taut high-tension vesicles, are opsonized with AlexaFluor647 anti-biotin and mixed with LifeAct-GFP-expressing macrophages. During phagocytosis, the macrophage forms a phagocytic cup, identifiable from enriched actin in a ring, that encircles the GUV. The macrophage fully engulfs the GUV within tens of seconds. (B) GUVs filled with a hypoosmotic sucrose solution (270 mOsm) are at low tension and can be ‘pushed’ by a macrophage, as shown by the position of the vesicle over time relative to the mid-plane at t=0s. (C). When low-tension GUVs are trogocytosed by a macrophage, punctate ‘bites’ can be observed within the macrophage. Scale bar is 5μm. (D-F) Time-lapse confocal microscopy images show interactions between macrophages (Lifeact-mScarlet3, pseudo-colored in green) and antibody-opsonized lymphoma cells (pseudo-colored in magenta). D) Example of a macrophage engulfing an entire target cell. E) Example of a macrophage pushing the target cell. F) Example of a macrophages trogocytosing a fragment of the target cell. Scale bars are 10μm. In simulations, (G) the complete engulfment of the target vesicle when its osmotic pressure is high $p=10\;k_{B}Tl_{\text{min}}^{-3}$. (H) The target is pushed when the osmotic pressure is intermediate $p=0.5\;k_{B}Tl_{\text{min}}^{-3}$. A plane that is shown perpendicular to the pushing direction through the middle of the target vesicle’s initial position. (I) For a very low osmotic pressure, $p=0.1\;k_{B}Tl_{\text{min}}^{-3}$, the cell-like vesicle takes a bite from the target.

In Fig.7 we compare the theoretical predictions of three dynamical phases as function of the membrane tension of the target vesicle, to experimental observations. Fig.7(A–C) gives examples of snapshots of the interactions between macrophage cells (labeled in green) and giant unilamellar vesicles that contain antibodies that trigger macrophage adhesion (GUVs, labeled in pink) [20]. In the experiments the membrane tension was varied using different sucrose solutions to fill the GUVs, and as function of decreasing membrane tension the observed behavior changes from (mostly) engulfment at high tension (Fig.7A), to a mixture of pushing and some biting at intermediate tensions (Fig.7B), finally exhibiting biting (trogocytosis) activity for the lowest GUV membrane tensions (Fig.7C, See Movies S2, S3, S4).

In Fig.7(D–F) we show examples of a macrophage (green) interacting with a lymphoma cell (magenta). From top to bottom we see the same three typical behaviors: engulfment (Fig.7D), pushing (Fig.7E) and biting (trogocytosis, Fig.7F) (See Movies S5, S6, S7 respectively). While we do not know exactly the stiffness of each of these cancer cells, finding this range of behaviors and comparing to our model suggests that cancer cells exhibit a wide range of stiffness [35]. This trait of cancer cells was indeed measured using Atomic Force Microscopy (AFM) [36, 37].

In Fig.7(G–I) we demonstrate how the three phases observed in the experiments are predicted by our theoretical simulations as a function of the pressure inside the engulfed target vesicle (See Movies S8, S9, S10, respectively). Higher internal pressure acts to suppress dynamic changes and fluctuations in the target vesicle’s area, equivalent to higher membrane tension (Fig.S11). Note that we do not allow our vesicles to undergo topological changes such as fission, so the biting behaviour in the simulations is arrested.

Our model also offers an explanation to the puzzling observations of cells either engulfing or pushing away apoptotic cells during embryogenesis [38]. Similar to our simulations, cells were observed to push away some apoptotic cells, and eventually detach from them, as we calculate (Fig.4D). We propose that the engulfed cells in this system are either stiffer, or happen to be confined by neighboring cells (Fig.S9).

## DISCUSSION

We have explored here the dynamics of cellular adhesion and engulfment of soft objects, whereby the mechanism of the self-organization of the protrusive forces of actin polymerization is through curved membrane protein complexes (CMC). Curved membrane proteins that recruit actin polymerization were shown to drive cellular protrusions during cell adhesion and migration [11, 13, 14]. This mechanism was previously shown to explain how cells phagocytose rigid objects [8], and here we explored this mechanism when the engulfed object is flexible.

Our model predicts that as function of the stiffness or membrane tension of the engulfed vesicle, there are distinct dynamical phases: While a stiff object is engulfed, a softer object will be spontaneously pushed away until it detaches. The softest objects get partially engulfed, which we expect in reality to result in a piece getting ”bitten” off (trogocytosis). The theoretical model explains the origin of these dynamical phases as arising due to the feedback between the deformations of the engulfed object, and the orientation of the active forces exerted by the cell’s leading edge. Comparing to experiments of immune cells engulfing artificial vesicles or cancer cells, we validate the predicted relation between engulfment dynamics and the mechanical deformability of the target.

Note that our model predicts that successful engulfment of soft targets is facilitated by the cell employing weaker cytoskeletal forces (Fig.3). Altogether, these results suggest possible future interventions to enhance or inhibit phagocytosis and trogocytosis based on the interplay between the target’s stiffness and the protrusive activity of the engulfing cell.

Our model is using only the most minimal physical components and forces, and therefore offers a path to obtaining deep and general understanding of an important biological process which is shared by many cell types [39–41]. Future extensions of our modeling approach could include additional processes, such as myosin-induced contractility and the effects of actin treadmilling-induced forces, in addition to more complex description of the adhesion between the cell and its target (which may it-self depend on stiffness and applied forces [20]).

## I. METHODS AND MATERIAL

### Lipids—

Phosphocholine (PC) lipids (Avanti Polar Lipids) were used as purchased without further purification. Lipid stock solutions in chloroform contained a ternary mixture of 98 mol% POPC, 1 mol% biotin-PE, and 1 mol% PEG2K DSPE. GUVs are diluted in an ionic solution of PBS and all lipids in our mixtures are zwitterionic. We added PEG2K DSPE to block GUVs from aggregating in the charge-screened PBS solution.

### Antibodies—

Antibodies used to opsonize GUVs were purchased from Santa Cruz Biotechnology and used without further labeling or purification. Biotin was bound by AlexaFluor647-labeled anti-biotin mouse IgG (clone BK-1/39, Santa Cruz Biotechnologies).

### RAW 264.7 cell culture—

RAW 264.7 murine male macrophage-like cell line was obtained from and authenticated by the UC Berkeley Cell Culture Facility. Cells were cultured in RPMI 1640 media (Corning) supplemented with 10% heat-inactivated fetal bovine serum (HI-FBS, Thermo Fisher Scientific) and 1% Pen-Strep (Thermo Fisher Scientific). RAWs were cultured in non-tissue culture-treated 10 cm dishes (VWR) at 37°C, 5% $CO_{2}$.

### Stable LifeAct GFP RAW 264.7 cell line—

HEK293T cells were grown in a 6-well plate to 80% confluency, and 160 ng VSV-G, 1.3μg CMV 8.91, and 1.5μg target pHR LifeAct GFP expression vector were transfected into HEK293T cells using TransIT-293T transfection reagent (Mirus Bio). Viral supernatants were collected 60 hours after transfection and spun at 4000 G to remove HEK293T cells. Viral supernatant was stored at 4°C for no longer than 48 hours prior to infection. For lentiviral infection, 500μL of viral supernatant was added to 5e5 RAW 264.7 macrophages along with 4μg/mL polybrene, and cells were spun at 400G for 25 minutes at 37°C and then resuspended and plated in a 6-well plate. Viral media was replaced with fresh growth media 24 h after infection. Cells were sorted via fluorescence-activated cell sorting on an Influx Cell Sorter (Beckton-Dickinson), and a population of cells expressing LifeAct GFP was expanded and frozen for later use.

### GUV electroformation—

Solutions containing 0.25 mg total lipids were spread evenly on slides coated with indium tin oxide (70-100Ω/sq; Sigma Aldrich). The slides were placed under vacuum for >30 min to allow for complete evaporation of chloroform. A capacitor was created by sandwiching a 0.3-mm rubber septum between two lipid-coated slides. The gap was filled with 200μL of 300 mM sucrose (hyperosmotic solution compared to PBS (285 mOsm) to make high-tension GUVs) or 270 mM sucrose (hypoosmotic solution compared to PBS to make low-tension GUVs). Sucrose solution osmolarity was measured using an osmometer (Precision Systems). GUVs 10 to 100μm in diameter were electroformed by application of an AC voltage of 1.5 V at 10 Hz across the capacitor for 1 h at 55°C.

### Imaging techniques—

All live cells were maintained at 37°C, 5% ${CO}_{2}$ with a stage top incubator (Okolab) during imaging. For confocal microscopy, cells were imaged with a spinning disk confocal microscope (Eclipse Ti, Nikon) with a spinning disk (Yokogawa CSU-X, Andor), sCMOS camera (Prime 95B, Photometrics), and a 60x objective (Apo TIRF, 1.49NA, oil, Nikon). The spinning disk confocal microscope was controlled with Nikon Elements (Nikon). Images were analyzed and prepared using FIJI (imagej.net/software/fiji).

### Phagocytosis/trogocytosis of GUVs—

50,000 macrophages were seeded in wells of an 8-well glass-bottom plate (CellVis) in 100μL of RPMI 1640 medium. Post-seeding, cells were incubated at 37°C, 5% ${CO}_{2}$ for 3–4 hours before target addition. 100μL of low-tension GUVs or high-tension GUVs (1 million GUVs counted with an impedance-based cell counter (Scepter, SigmaAldrich)) were prepared with 4μM AlexaFluor647 anti-biotin IgG in PBS and allowed to incubate with gentle rotation for > 10 minutes. After washing, GUVs were added to macrophage-seeded wells on the stage top incubator of the microscope.

### Time-lapse confocal microscopy of antibody-dependent cellular phagocytosis —

J774A.1 macrophages and Raji B lymphocytes were obtained from DSMZ (ACC170, ACC319). J774A.1 that stably express Lifeact-mScarlet31 [42] were cultured at 37°C with 5% ${CO}_{2}$ in DMEM (Wisent, 319–005) supplemented with 10% of heat-inactivated FBS (Wisent, 090–150). Raji were cultured were cultured at 37°C with 5% $CO_{2}$ in RPMI-1640 (Wisent, 350–007) supplemented with 10% of heat-inactivated FBS (Wisent, 090–150). 24 hours before imaging, 100,000 macrophages were seeded into 18 mm circular #1.5 coverslips. Prior to the experiment, Raji cells were stained with Calcein AM viability dye (eBioscience, 65-0853-78) at 1μM for 30 minutes. Macrophages were transferred to a Chamlide CMB imaging chamber (Live Cell Instrument) and incubated in DMEM without phenol red, containing 25 mM HEPES (Wisent, 319–066). 200,000 Raji cells were added to the macrophages, and 2μg/ml of anti-human CD20 antibody (BioXcell, Rituximab) and 10μg/ml of anti-human CD47 antibody (BioXcell, B6.H12) were supplemented. Images were acquired every 20 seconds for 2 hours using a 40x 1.3 NA oil immersion objective on a Nikon Eclipse Ti2-E, equipped with a Yokogawa SCU-W1 spinning disk, a Hamamatsu Orca-Fusion BT sCMOS camera, and a stage-top incubator (Tokai Hit) to maintain cells at 37°C. Images were acquired using Nikon NIS Elements and analyzed using Fiji is just ImageJ 1.54p.

### Microparticle traction force microscopy (MP-TFM) analysis of phagocytosis of DAAM-particles—

Experimental data was obtained from prior conducted experiments [43] in which RAW 264.7 macrophages were exposed to IgG-functionalized 1.3 kPa deformable poly-Aam-co-AAc microparticles (DAAM-particles), stained for F-actin using Alexa Fluor-488 conjugated phalloidin, and imaged by confocal microscopy [44]. DAAM-particle 3D shape reconstructions and force analysis were performed as previously described [18, 45]. Briefly, the inverse problem of inferring the traction forces T is solved iteratively until a minimal gradient tolerance is reached. During this optimization, an ideal sphere with the same particle volume of the measured individual particle is subjected to a trial displacement field (u) to exactly match the surface of the experimentally observed shape of the DAAM-particle, while minimizing the cost function:

(3) $$f(u)=E_{el}+\alpha R^{2}\left(T;\partial \Omega_{t}\right)+\beta E_{pen}(T)$$

where R(T;∂Ωt) represents the residual cellular forces exerted outside of the cell-target contact region, defined from the phalloidin and immunostaining. The elastic energy ($E_{el}$) penalizes unphysical solutions in which larger forces producing the same shape, while $\beta E_{\text{pen}}$ serves as an anti-aliasing term. The weighing parameters, a(residual traction) and β(anti-aliasing), were both set to 1. Spherical harmonic coefficients up to $l_{\text{max}}=20$ were utilized and normal forces were evaluated on a 21 × 41 grid.

### Interaction between the vesicles—

Two vesicles were left to interact through adhesion. The adhesive energy per node between two vesicles is set to $E_{\text{ad}}=2k_{B}T$. If the vertices from different vesicles come within the interaction range (set to $l_{\text{min}}$) then this adhesive energy is taken into account. The process is passive when the interaction is solely through the adhesion between them, and there is no active force imparted on the target vesicle by the bigger cell-like vesicle. An active force is imparted by a CMC node in the cell-like vesicle on a vertex in the target vesicle. To calculate the tangential and normal force imparted, we calculate the interaction force first. Let $V_{i}$ be the vertex of interest in the target vesicle, and ${\overset{ˆ}{n}}_{i}$ be its outward normal (computed by taking the average of the normals for all the triangles having the vertex in common). The normal and tangential components of the interaction force due to the sum of active forces acting on this vertex, $F_{i}$, are given by,

(4) $$\begin{array}{r} F_{⊥}=\left(F_{i}\cdot {\overset{ˆ}{n}}_{i}\right){\overset{ˆ}{n}}_{i},\\ F_{\|}=F_{i}-\left(F_{i}\cdot {\overset{ˆ}{n}}_{i}\right){\overset{ˆ}{n}}_{i}. \end{array}$$

For a more detailed description, see SI sections S1–S3 and the methods and material.

### Setup for the engulfment simulation—

In all the simulations of the engulfment process, we construct two vesicles: i) a big cell-like vesicle with 3127 vertices, out of which 150 vertices represent the curved proteins with intrinsic mean curvature $c_{0}=1l_{\text{min}}^{-1}$, the protein-protein interaction energy is set to $w=1\;k_{B}T$, and ii) a smaller target vesicle made of 847 vertices with no curved proteins. At the initial time, the two vesicles were nearly spherical in shape and placed very near to each other, within the adhesion distance. The bending rigidity for the cell-like vesicle is set to $20k_{B}T$ throughout the paper. We varied the bending rigidity of the target vesicle from $20k_{B}T$ to a very high value of $2000k_{B}T$, which is nearly a rigid sphere.

## Supplementary Material

1

## Data Availability

The MATLAB code for analyzing confocal images and deriving particle shape is publicly available on https://gitlab.com/dvorselen/DAAMparticle_Shape_Analysis The Python code for traction force analysis is available on https://gitlab.com/micronano_public/ShElastic.

## Significance statement—

Phagocytosis is essential for immune defence, yet the physical principles governing the engulfment of soft, deformable targets remain poorly understood. Most theoretical models assume rigid particles, even though real cells undergo substantial shape changes during contact. Here, we develop a theoretical membrane model to simulate interactions between multiple vesicles, enabling a mechanistic exploration of phagocytosis of soft targets. We show that target membrane rigidity dictates whether it is fully engulfed, pushed away, or partially bitten. These mechanically driven regimes explain experimental observations of immune cells engaging with both artificial GUVs and lymphoma cells.
